# Patient value: Perspectives from the advocacy community

**DOI:** 10.1111/hex.12628

**Published:** 2017-09-20

**Authors:** Bonnie J Addario, Ana Fadich, Jesme Fox, Linda Krebs, Deborah Maskens, Kathy Oliver, Erin Schwartz, Gilliosa Spurrier‐Bernard, Timothy Turnham

**Affiliations:** ^1^ Bonnie J Addario Lung Cancer Foundation (ALCF) San Carlos CA USA; ^2^ Men's Health Network Washington DC USA; ^3^ Roy Castle Lung Cancer Foundation Liverpool UK; ^4^ International Society of Nurses in Cancer Care Vancouver BC Canada; ^5^ International Kidney Cancer Coalition (IKCC) Co‐founder Kidney Cancer Canada Guelph Ontario Canada; ^6^ International Brain Tumour Alliance (IBTA) Surrey UK; ^7^ Strategic Partnerships and Communications The Max Foundation Seattle WA USA; ^8^ Melanoma France Teilhet France; ^9^ Melanoma Research Foundation Washington DC USA

**Keywords:** patient‐derived preferences, patient‐relevant values, patient‐reported outcomes, value frameworks

## Abstract

All health‐care systems are under financial pressure and many have therefore developed value frameworks to assist decision making regarding access to treatment. Unfortunately, many frameworks simply reflect the clinically focused values held by health‐care professionals rather than outcomes that also matter to patients. It is difficult to define one single homogeneous set of patient values as these are shaped by social, religious and cultural factors, and health‐care environment, as well as many factors such as age, gender, education, family and friends and personal finances. Instead of focusing on an aggregated set of values, frameworks should attempt to incorporate the broader range of outcomes that patients may regard as more relevant. Patient advocates are well placed to advise assessment bodies on how particular therapies will impact the patient population under consideration and should be closely involved in developing value frameworks. In this paper, a group of patient advocates explore the varying definitions of patient value and make positive recommendations for working together to strengthen the patient voice in this area. The authors call on framework developers, the patient advocacy and research communities, the health‐care industry and decision‐makers to undertake specific actions to ensure patient value is included in current and future value frameworks. This is justified on compassionate and economic grounds: better health outcomes result when patients receive treatment tailored to individual needs. Paying attention to the patient perspective also results in better use of resources—a goal that should appeal to all stakeholders.

## 1 | INTRODUCTION

In all health-care systems around the world, decisions regarding access to treatment have become more complex due to budgetary pressures: static or declining health-care budgets mean that stakeholders may be competing against each other for diminishing resources.

Treatment decisions have been further complicated—particularly in the field of cancer—by the increasing number of treatment options for a single disease with each offering a different set of trade-offs between cost, benefit and risk.[1,2] These practical concerns have largely contributed to the recent interest in value frameworks,[3] which are tools used to assess the "value" of treatment based on calculating the cost of various care options and comparing them to the anticipated health benefit, as assessed through clinical trials. Although based on differing methodologies and with different stated aims,[4] they are intended to guide the allocation of resources fairly and equitably[5] as all stakeholders agree on the goal of improving outcomes and doing so as efficiently as possible.

The European Society of Medical Oncology (ESMO) published its framework in 2015,[6] and a number of frameworks have also been proposed in the USA to support decisions in cancer care, for example from the American Society of Clinical Oncology (ASCO),[7] the Institute for Clinical and Economic Review (ICER),[8] Memorial Sloan Kettering Cancer Center,[9] and the National Comprehensive Cancer Network (NCCN),[10] and in the field of cardiology.[11]

However, the value judgements incorporated into these frameworks very often reflect the standard economic and clinical attributes that decision-making bodies have previously defined. While this has obvious relevance to patient well-being, this approach does not necessarily address all the values that are important to patients. If patients are to receive the treatment that they feel is best for them, clinical guidelines and decision-making frameworks clearly need to be built on a thorough understanding of the patient perspective rather than on long-standing practice, or the views of treating clinicians or the panel of experts involved in developing frameworks.

Patient advocates have an important role to play here as they are aware of and can express many viewpoints of the patients they represent. In this paper, a group of patient advocates representing many thousands of patients from around the world explore the following:

- The varying definitions of patient value
- Value from the patient perspective
- Why and how decision-makers should incorporate patient value in frameworks and guidelines
- How the advocacy community can work together to ensure that the concept of patient value is included in current and future value frameworks.

Throughout the paper, in the interests of brevity, we refer to "patients" but, as many patients rely on their caregivers to act in their best interests, this should be taken to mean "patients and their care-givers." Also, although the patient advocates involved in this paper represent cancer patients, the issues and principles laid out here apply equally well to other patients, particularly those with chronic, degenerative or life-threatening illnesses, where decisions about care can be particularly preference-sensitive.

## 2 | THE CHANGING VIEW OF PATIENT VALUES

The clinician has historically been at the centre of decision making, basing decisions for the patient on whether a treatment was "clinically relevant." Before the knowledge gap between clinicians and patients became smaller, many patients were complicit with this and were happy to leave treatment decisions to their doctors.[12]

However, this paternal approach to decision making may misrepresent how patients value the benefits and risks of various treatment options. Clinicians are strongly influenced by the scientific tradition of medicine, focusing on factors that are objectively measurable. If a particular treatment has a measurable physiological effect, then it is natural to assume that the patient will feel better and live longer.[12]

Clinicians are trained to consider how much benefit a treatment can offer before it is worth subjecting a patient to its side-effects, long-term risks, inconveniences and costs. The drawbacks of this clinician-led approach are highlighted in a review of the literature relating to decision making in lung cancer that found wide disagreement between patients and doctors in the perceived value of chemotherapy.[13] There was a strong mismatch in the degree to which patients and clinicians valued survival, with patients placing a higher value on survival while clinicians emphasized factors such as toxicity and symptoms.[13,14]

Leaving decision making to the clinician is further complicated by the fact that clinicians will themselves make value judgements that differ from those of their colleagues, let alone their patients.[12] When asked to recommend treatment for the same critically ill patient, one clinician may argue for withdrawal of life support, while another may recommend a programme of aggressive intervention.[15]

## 3 | PATIENT VALUES FROM THE PATIENT PERSPECTIVE

The habit of viewing treatments entirely through the lens of clinical relevance has been based on the assumption that patients share the same set of values as clinicians and decision-makers, with a focus on survival, balanced by a consideration of toxicity and side-effects.[16] The reality is much more complex.

Clinical evidence typically takes the form of probabilities applicable to populations, not individual patients; while patients' understandings are individual, idiosyncratic and based on personal experience, interpretation of science and other factors.[17] At a population level, patient values are shaped by social, religious and cultural factors and the health-care environment in which patients live (see Box S1 for examples). At an individual level, treatment-based values are important but are also influenced by age, gender, education,[18] family and friends, attitude to work and career, personal finances, amongst others.

Furthermore, values are not static, but will change as the patient's individual circumstances and the experience of illness and treatment change throughout his or her disease journey, reflecting shifts in prognosis, severity of disease, comorbidities, available treatment or palliative options and life events. Rarely acknowledged in formal frameworks is the value that patients may place on non-interventional support such as access to patient support groups, clinical nurse specialists, palliative care, rehabilitation, and so on.

There are examples in the literature from around the world showing how values differ between groups, individuals and over time. For instance, Korean Americans, Mexican Americans, African Americans and Hispanic patients are more likely to believe that the family should be involved in making decisions about treatment, while Caucasians may be more likely to value individualistic beliefs such as self-reliance, responsibility and control.[19,20]

Some studies have found that older and less educated individuals were more likely to prefer decisions to be made for them, while younger, and more educated patients preferred to participate in decision making.[21-26] The dynamic nature of patient preference has been illustrated in a study that found a decline in patients' desire for involvement as they became more ill.[27]

In a cross-sectional survey, patients with colorectal cancer ranked family preference, family burden and travelling for treatment as important factors influencing their treatment decisions[28] yet these and other factors are rarely, if ever, included in frameworks or guidelines.

In summary, patient values clearly do not always accord with traditionally defined health-seeking goals.[29] Patients rarely make a straightforward trade-off between survival and side-effects but are influenced by a wide variety of internal and contextual factors. This tension is reflected in the way in which value frameworks are developed: some health-care systems, when they do take input from patients and their advocates, focus on societal values and benefits as the important criteria to consider when making decisions, while others prioritize the individual values and benefits that matter to a single patient.

## 4 | WHY PATIENT VALUES SHOULD BE REFLECTED IN FRAMEWORKS

Existing frameworks are largely constructed through an evidence-based medicine (EBM) approach that relies on published data from RCTs, while excluding real-world evidence (RWE, based on data collected from sources outside of clinical trials such as observational or registry studies, retrospective database collection and case reports and reports of patient experience), to demonstrate an improvement in outcomes. Care plans that focus on individual needs are not necessarily in conflict with this approach: the EBM approach proposes that patients and clinicians "make a choice together after considering the best available evidence, the clinician's experience and *the patient's values*."[30]

There is substantial evidence to show that patient-centred care improves patient satisfaction, adherence to medications, improved chronic disease control, quality of care and health outcomes while reducing costs and disparities in health care.[31,32] It is now largely accepted that a "good" outcome must be defined in terms that are meaningful and valuable to the individual patient[12,33] and value in health care should be measured by the outcomes achieved, not by the volume of services delivered.[5] As many insurance-based systems, not just those in the US, are attempting to move away from a fee-for-service model, the notion of value—particularly as experienced by the patient—should be an attractive concept.

An advisory report to the World Health Organization states that "values should always be considered in making recommendations….."[34] As differences in patient preferences may lead to differences in the preferred therapy, a guideline that does not consider these preferences may provide recommendations that are suboptimal.[35]

Although frameworks need to incorporate data on the outcomes that matter to patients, very often they simply reflect the more clinically focused values held by clinicians and other health-care professionals. It has been reported that most guidelines do not systematically seek or integrate evidence on patient preferences. Chong et al[36] found that less than 5% of the text and references in high-quality guidelines from a variety of disciplines was devoted to patient preferences, compared to nearly 25% of text dedicated to treatment effectiveness. Half of the guidelines studied did not cite any preference-related evidence at all.

Patient input is necessary to determine the relative importance of the full range of outcomes that patients may experience, rather than the limited set of outcomes considered relevant by researchers, clinicians and health-care professionals. For example, treatment burden may not seem to be a critical factor to a young person with a potentially lethal cancer that is still curable; survival is more likely to be the key consideration. On the other hand, treatment burden, quality of life and function are likely to be much more important to a frail and elderly patient. Both of these differing sets of values demand to be reflected in the treatment options made available. Guidelines panels, decision-makers and committees should not assume that patient values are uniform and unchanging across patient populations.[37] As Silvestri et al[38] commented: when different patients value alternative treatment options differently, clinical rules or protocols based on average preferences will not be effective in helping patients select the treatment they actually want.

## 5 | THE PROVIDER VIEW OF VALUE FRAMEWORKS

A recent survey carried out on 11 health plans in the USA found that most do not use existing value frameworks to make coverage decisions and are not likely to do so in the next year.[39] One factor contributing to this decision was the need for a consideration of the total treatment costs involved with a particular therapy, rather than just the drug cost. To reduce costs, the best approach is often to spend more time or money on some services to reduce the need for others:[5] high short-term costs may reduce overall health costs down the line if outcomes are better or if there is less need for collateral interventions.

Interestingly, the concept of including the patient perspective into value frameworks appears to resonate with many health plans. Another reason given for not using existing frameworks was that they felt frameworks needed to integrate broader perspectives of value—*especially the patient view*— to gain greater acceptance.[39] It is felt that, while current frameworks lay the ground work for a push towards value, "there is still room for improvement."

Studies carried out in Europe and elsewhere indicate that non-adherence to guidelines is common when clinicians feel that patients' individual circumstances mean they will not be best served by following the standard recommendations.[40]

Incorporation of patient values into decision making will not necessarily lead to demands for treatments "at any cost." A 1997 paper claimed that cancer patients were "cost-insensitive."[41] However, other studies have demonstrated that patients do not always demand extended survival at any price. In a "trade-off" exercise in patients with non-small cell lung cancer (NSCLC), 57% would choose chemotherapy only if the one-year survival offered a minimum of a 10% advantage over other treatments.[42]

A systematic review of treatment preferences for metastatic and locally advanced NSCLC found a wide variation in how patients viewed survival benefits: while some patients would regard an increased survival rate of 1% as sufficient, others would require survival rate to increase by 50%, and a third group did not regard chemotherapy as worthwhile under any circumstances. As the authors concluded: the survival benefits patients require to make particular treatments worthwhile were "moderate, widely variable and difficult to predict."[43]

These survival benefits are also conditional upon many other factors, particularly the severity of disease symptoms: the value of improvement in progression-free survival is usually conditional upon control of disease symptoms rather than being a goal in itself.[44]

In one of the few studies to also include the cost of treatment in their analysis, cancer patients in an insurance-based health-care system expressed specific preferences for survival and low toxicity but wished to avoid very large out-of-pocket costs. Unfortunately, this was more of an issue for patients with lower socioeconomic status who were more likely to choose cheaper treatments even if these came with lower survival or higher toxicity.[45] An analysis of patients with early stage breast cancer found that patients who faced high co-payments for adjuvant hormonal therapy are less compliant with therapy.[46]

It is also the case that many patients value services such as physiotherapy and nursing support, which can be provided at relatively low cost compared to some interventional treatments.

## 6 | THE ROLE OF THE PATIENT ADVOCACY COMMUNITY IN FRAMEWORK DEVELOPMENT AND DECISION MAKING

All advocacy groups share the same goal, which is to include the patient perspective in the frameworks and decision-making processes that are drawn up by those who determine access to medicines. As patient advocates, we have a responsibility to understand the differences between health systems and to talk about the values that reflect our individual patient communities. We should also attempt to understand the motivations and concerns of those patients who do not wish to be part of, or represented by, a formal advocacy group.

To achieve this, we must be clearer and more focused about what we mean when talking about patient value. Advocates have different perspectives of value depending on the patient groups that they represent but despite these differences, the most important role for advocates is to work together to ensure that appropriate choice and flexibility is retained in the available treatment options. Patient advocates are well placed to provide a picture to assessment bodies of how particular therapies would impact the patient population under consideration and to encourage negotiation on costs.

Advocacy groups are already discussing how to work together to build the evidence for the values we are promoting. One challenge is to work out how to gather evidence that demonstrates the various aspects of patient value and to develop methodology to quantify them. Attempts are also being made to develop a taxonomy that is different to, but parallel with the RCTs and the meta-analyses used in conventional clinical assessments while new trial designs are emerging that may be more reflective of patient-relevant outcomes.

## 7 | PRACTICAL STEPS TO INCORPORATING THE PATIENT PERSPECTIVE INTO VALUE FRAMEWORKS

In this paper, we have demonstrated that the incorporation of patient values into value frameworks is justified not just on moral grounds but also on economic grounds: numerous studies have shown that better health outcomes can be achieved with patient-centred care.

However, the patient perspective cannot be inferred by expert panels but needs to be provided by patients and those advocacy groups who speak for countless numbers of patients around the world. We also need to remember those patients who are inherently less proactive in their care, and who may not be represented by patient advocacy groups. As a result, formal studies and the perspectives of patient advocates may not fully incorporate experiences or viewpoints of the broader patient population. Such patients are more difficult to identify, but their understanding of value is equally important.

As advocates, we believe it is possible to incorporate into frameworks the aggregated values that matter to patients across different disease cohorts, and the detailed and particular values that matter at the very personal level to an individual patient. Despite the varying nature of patient values, this need not be a daunting or difficult task. We propose the following steps:

### 7.1 | Benefit

There needs to be agreement that including patient preferences and values in frameworks and guidelines benefits clinical practice.[36] Although this may add another level of complexity, to ignore these data would be to systematically exclude one very important class of relevant evidence. While the range of patient values may seem to be overwhelmingly complex, researchers in this field have cautioned decision-makers against becoming paralysed by complexity and uncertainty, to achieve more meaningful outcomes.[47]

### 7.2 | Representation

Montori[37] recommends that guidelines panels—the same applies to framework developers—should include representatives of the target users, which means patients as well as clinicians. Patients and their advocates should be present from the start of the process and should not simply be brought in at the end. There should be representation from a collection of advocates, not just a token patient voice, to ensure that the frameworks acknowledge a range of values that are important to patients. This is already happening in the area of kidney cancer but needs to become commonplace rather than the exception.[48] All involved should also seek ways to elucidate information from patients who are not being treated at major academic centres and are not actively engaged with patient advocacy groups.

### 7.3 | Evidence

The panels and committees that develop these value frameworks need to evaluate and incorporate published research on patient preference. Scientific studies of patient preferences are more common than is generally acknowledged and are a valid means of understanding general trends in patient values and of identifying decision points that may be particularly preference-sensitive.[36]

### 7.4 | Language

The use of more appropriate language may help to condition the way developers think when constructing frameworks. A shift from considering what is "clinically relevant" to what is "patient important" or of "personal significance" could result in a very different set of recommendations being made.[12]

## 8 | CALL TO ACTION

In general, decision-makers and value framework groups are still failing to fully integrate patient values—whether aggregated or individual—in existing frameworks and health-care resource decisions. In some areas, this is due to deficiencies in identifying and categorizing patient values but much research exists which demonstrates the importance of patient values in health-care provision.

*We call on framework developers* to bring a broad panel of patient advocates, with a selection of perspectives covering a range of disease types, patient demographics, prognosis expectations and treatment options into framework development committees. For frameworks that have already been launched, we call on the development committees to include a similarly broad group of advocates as equal members in their continuing evolution of these tools to ensure that they are truly capturing the relevant domains of patient value.

*We call on the patient advocacy community* to further strengthen the narrative and evidence based on patient values to make sure that patient values capture both the societal needs of patient cohorts alongside the values of individual patients within the context of each disease community and health-care environment.

*We call on the research community* to advance methodologies for recording, quantifying and assessing patient values, including in RCTs, and sharing these methodologies through publication to build an evidence base.

*We call on the health-care industry* to work more closely with the advocacy community to take an active role in PRO (patient-reported outcomes) development, ensuring that new tools contain the domains that our patient members tell us are important. Patient-reported outcomes tools are increasingly being used in clinical studies as a measure of patient experience, but the development of meaningful PRO tools will only be possible with adequate involvement of patients and advocates from the beginning. We also call on the industry to invest in the generation of patient evidence in partnership with the advocacy communities.

*We call on decision-makers* such as HTA and payer bodies to develop processes within their decision making that bring the patient perspective into these decisions. We urgently call on these decision-makers to ensure that patients have equal representation on decision-making committees alongside clinical and health economics experts. No health-care resource decision should be made without including and considering the patient perspective.

Furthermore, we call on HTA and payer bodies to develop specific expertise and skill sets to assess qualitative evidence so that this evidence is not devalued when viewed alongside clinical and economic evidence.

Finally, we call on all HTA bodies to sign up to the HTAi values and standards of patient involvement in HTA to ensure that their boards and management are measured by the degree to which they involve patients, advocates and citizens in the HTA decisions they make.

## 9 | CONCLUSION

Budgetary pressures have contributed to the rise of HTA assessments and the recent interest in developing value frameworks. However, the value judgements incorporated into these assessments and frameworks rarely reflect the values that are important to patients. Patient input is necessary to define the response to the full range of outcomes that patients may experience, whether this is at an aggregated or a personal level, rather than the limited set of outcomes considered relevant by researchers. The patient perspective cannot be inferred by expert panels but needs to be provided by patients and advocacy groups. We have proposed a set of steps for framework developers to follow to fully incorporate the patient perspective into their guidance.

As advocates for the patient, we believe that this approach is mandated not just on compassionate grounds but also for sound economic reasons. As has been shown time and again, paying attention to the patient perspective results in better health outcomes and better use of resources. This is a goal that should appeal to all stakeholders in health care.

As patient advocates, we are willing to engage in dialogue with any group involved in developing a value framework to ensure that the resulting guidance is strengthened through the inclusion of patient values.

### ACKNOWLEDGEMENTS

Jude Douglass, Healthcom Partners Ltd, Oxford, United Kingdom for assistance in writing and editing the manuscript with financial support from Bristol-Myers Squibb. Bristol-Myers Squibb had no role in drafting or approving the manuscript.

## SUPPORTING INFORMATION

Additional Supporting Information may be found online in the supporting information tab for this article.

**How to cite this article:** Addario B, Fadich A, Fox J., et al. Patient value: Perspectives from the advocacy community. *Health Expect*. 2018;21:57–63. https://doi.org/10.1111/hex.12628
